# Putative functions of extracellular matrix glycoproteins in secondary palate morphogenesis

**DOI:** 10.3389/fphys.2012.00377

**Published:** 2012-09-24

**Authors:** Rocca d'Amaro, Rolf Scheidegger, Susan Blumer, Pawel Pazera, Christos Katsaros, Daniel Graf, Matthias Chiquet

**Affiliations:** ^1^Department of Orthodontics and Dentofacial Orthopedics, School of Dental Medicine, University of BernBern, Switzerland; ^2^Institute for Oral Biology, Center for Dental Medicine, University of ZurichZurich, Switzerland

**Keywords:** palate morphogenesis, growth factors, transforming growth factor beta, bone morphogenetic protein, extracellular matrix, fibronectin, tenascin, fibrillin1

## Abstract

Cleft palate is a common birth defect in humans. Elevation and fusion of paired palatal shelves are coordinated by growth and transcription factors, and mutations in these can cause malformations. Among the effector genes for growth factor signaling are extracellular matrix (ECM) glycoproteins. These provide substrates for cell adhesion (e.g., fibronectin, tenascins), but also regulate growth factor availability (e.g., fibrillins). Cleft palate in *Bmp7* null mouse embryos is caused by a delay in palatal shelf elevation. In contrast, palatal shelves of *Tgf-β3* knockout mice elevate normally, but a cleft develops due to their failure to fuse. However, nothing is known about a possible functional interaction between specific ECM proteins and Tgf-β/Bmp family members in palatogenesis. To start addressing this question, we studied the mRNA and protein distribution of relevant ECM components during secondary palate development, and compared it to growth factor expression in wildtypewild type and mutant mice. We found that *fibrillin-2* (but not *fibrillin-1*) mRNA appeared in the mesenchyme of elevated palatal shelves adjacent to the midline epithelial cells, which were positive for *Tgf-β3* mRNA. Moreover, midline epithelial cells started expressing fibronectin upon contact of the two palatal shelves. These findings support the hypothesis that fibrillin-2 and fibronectin are involved in regulating the activity of Tgf-β3 at the fusing midline. In addition, we observed that tenascin-W (but not tenascin-C) was misexpressed in palatal shelves of Bmp7-deficient mouse embryos. In contrast to tenascin-C, tenascin-W secretion was strongly induced by Bmp7 in embryonic cranial fibroblasts *in vitro*. These results are consistent with a putative function for tenascin-W as a target of Bmp7 signaling during palate elevation. Our results indicate that distinct ECM proteins are important for morphogenesis of the secondary palate, both as downstream effectors and as regulators of Tgf-β/Bmp activity.

## Introduction

In mammals, morphogenesis of the secondary palate starts with the formation of two palatal shelves that grow out ventrally from the maxillary processes on both sides of the tongue (Meng et al., 2009; Iwata et al., 2011). On embryonic day 14–15 in the mouse and in the ninth week of pregnancy in humans, the two vertically oriented palatal shelf anlagen rapidly elevate into a horizontal position, followed by their apposition at the midline. Later, the epithelia of two matching palatal shelves disintegrate in the midline, leading to the fusion of their mesenchymal compartments. In mouse mutant embryos, cleft palate can be caused either directly by disturbed growth, elevation or fusion of the palatal shelves, or indirectly, e.g., by malformations or impaired motility of the lower jaw and the tongue (Meng et al., 2009; Iwata et al., 2011). Malformations of the upper lip and the secondary palate are among the most frequent birth defects in humans (Mossey et al., 2009). In a high percentage the causes remain unknown, but both environmental (teratogenic) and hereditary factors might contribute (Meng et al., 2009; Iwata et al., 2011). An increasing number of regulatory genes have been linked to cleft lip and palate (CLP) in humans. For example, dominant mutations of transcription factor *IRF6* are responsible for Van der Woude Syndrome, whereas recessive mutations in the same gene cause non-syndromic cleft palate (Kondo et al., 2002). In other cases of CLP in humans, mutations have been found, e.g., in the genes for homeobox transcription factor *MSX1* (Alappat et al., 2003), and very recently for growth factor *BMP7* (Wyatt et al., 2010). Genetic studies have linked polymorphisms in the human genes for growth factors *TGF-α* and *Tgf-β3*, nuclear receptor *RAR-α*, and growth factor receptor *FGFR1* to CLP (Mossey et al., 2009). As expected, mice deficient for *Irf6*, *Msx1*, *Tgf-β3*, or *Bmp7* partially or completely mimic the cleft palate phenotype of humans with mutations or polymorphisms in these genes (Satokata and Maas, 1994; Proetzel et al., 1995; Ingraham et al., 2006; Zouvelou et al., 2009a). Concerning the mechanism of cleft palate formation, it is known that Tgf-β3 is required for the fusion of the palatal shelves (Proetzel et al., 1995; Taya et al., 1999). Whereas earlier work indicated that this growth factor stimulates epithelial-mesenchymal transformation at the palatal midline via transcription factors Smad2f/Lef1 (Nawshad and Hay, 2003), more recent evidence shows that Tgf-β3 signaling mediates palatal fusion mainly or exclusively by inducing apoptosis of middle edge epithelial (MEE) cells (Xu et al., 2006; Nawshad, 2008; Huang et al., 2011). In contrast, in embryos deficient for *Bmp7*, another member of the Tgf-β family, the elevation of palatal shelves seems to be disturbed (Zouvelou et al., 2009a), pointing to a defect in cell division, reorganization, and/or ECM production. For both human and mouse malformations, however, practically nothing is known about the downstream effectors of the mutated genes. Thus, the cellular mechanisms by which these regulatory genes finally cause a specific morphogenetic defect are not known as yet.

Extracellular matrix (ECM) is essential for tissue integrity by forming a stable framework to which cells adhere via integrin receptors (Alberts et al., 2008). During development and regeneration, ECM is constantly remodeled by growth factor controlled synthesis and proteolysis by matrix-degrading enzymes (Mott and Werb, 2004; Larsen et al., 2006). Whereas fibrillar collagens and large proteoglycans are major structural entities of ECM, more minor ECM components have important regulatory functions: they promote cell adhesion (fibronectin, laminin) (Alberts et al., 2008), modulate cell spreading and motility (tenascins) (Chiquet-Ehrismann and Chiquet, 2003), control collagen matrix assembly (FACIT collagens) (Zhang et al., 2005), or store growth factors and present them to cells (fibrillins) (Kaartinen and Warburton, 2003). Not much has been published yet about the function of ECM in secondary palate formation, although early reports recognized its importance (Morris-Wiman and Brinkley, 1992). Tenascin-C was implied due to its striking expression pattern during palatogenesis (Ferguson, 1988), but these studies were not continued. Other early work tried to establish a causal link between elevation of the palatal shelves and a rapid accumulation of hyaluronan (reviewed in Ferguson, 1988), but again these studies were not pursued. A more recent paper implied a Tgf-β3-induced chondroitin sulphate proteoglycan in palatal shelf adhesion, but its exact molecular identity was not determined (Gato et al., 2002). Thus, surprisingly little is known about the functional role of ECM in palatogenesis.

ECM genes are important transcriptional targets for Tgf-β/Bmp family members (Verrecchia and Mauviel, 2007; Tucker and Chiquet-Ehrismann, 2009). Interestingly, different members of the Tgf-β family can regulate even closely related ECM proteins in distinct ways. For example, tenascin-C expression is induced by Tgf-β1 but not Bmp2, whereas tenascin-W is regulated in the opposite manner (Tucker and Chiquet-Ehrismann, 2009), indicating that growth factor signaling can affect the balance of local ECM composition. Tenascins are structurally related to fibronectin, but have a quite opposite mode of action: whereas fibronectin promotes cell adhesion (Alberts et al., 2008), tenascins interfere with fibronectin function (Chiquet-Ehrismann et al., 1988; Huang et al., 2001). Thus, tenascins are anti-adhesive and pro-migratory, and their aberrant regulation due to the lack of a specific growth factor might change cell-ECM interactions and induce a specific malformation. To give another example, certain ECM components are important for the presentation and bioavailability of growth factors, e.g., fibrillins (Kaartinen and Warburton, 2003) and fibronectin (Dallas et al., 2005; Fontana et al., 2005) for Tgf-βs and Bmps, or proteoglycans for fibroblast growth factors (Iozzo et al., 2009). Faulty expression of an ECM component in the absence of one growth factor might thus disturb signaling by others.

However, a physical or functional interaction between Tgf-β/Bmp growth factors and specific ECM components during morphogenesis of the secondary palate has not been studied so far. In a first step to address this question, we describe here the specific expression patterns of *fibrillin-1*, *fibrillin-2*, *fibronectin*, *tenascin-C*, and *tenascin-W* mRNAs during craniofacial development of mouse embryos between E13.5 and E15.5 using *in situ* hybridization, and compare them to those of *Tgf-β1* and *Tgf-β3*.

## Materials and methods

### Animals, embryonic tissue, and cryosectioning

C57BL/6 wildtype mouse embryos were obtained from J.-F. Spetz at the Friedrich-Miescher Institute for Biomedical Research in Basel, Switzerland. The *Bmp7* null allele of *Bmp7* heterozygous null mice was generated by Cre-mediated recombination in the germ line of a conditional *Bmp7* allele (*Bmp7*^flx^), in which exon 1 is flanked by loxP sites as described earlier (Zouvelou et al., 2009b). *Bmp7* heterozygous null mice were intercrossed to obtain *B*mp7^+/+^, *B*mp7^+/−^ and *B*mp7^−/−^ embryos from the same litter. After mating, appearance of a vaginal plug was taken as embryonic day 0.5 (E0.5). Pregnant females were sacrificed by cervical dislocation at the desired stage (E13.5–E14.5), embryos were removed from the uterus and decapitated. Genotyping of embryos was carried out by allele-specific PCR. All procedures were approved by the Cantonal Veterinary Offices of Basel and Zurich, Switzerland. The embryo heads were washed in ice-cold phosphate buffered saline (PBS; 150 mM NaCl, 20 mM Na-phosphate, pH 7.4), fixed in 4% paraformaldehyde in PBS overnight, washed briefly in PBS, soaked for 24 h in 30% sucrose in PBS, embedded in Tissue Tek (O.C.T. compound; Sakura Finetek Europe B.V., Zoeterwoude, Netherlands), and frozen on a metal block cooled to −80°C. All tissue was stored at −80°C before sectioning. Serial frontal sections (10–12 μm thick) of the embryo heads were prepared on a Cryocut E cryomicrotome (Reichert-Jung, Leica Microsystems, Heerbrugg, Switzerland), dried at 37°C for 1–5 min, and stored at −80°C before further use.

### Gene-specific RNA probes and *in situ* hybridization

Total RNA was isolated from E14.5 C57BL/6 wildtype mouse embryos or from mouse embryo fibroblasts (Maier et al., 2008) using an RNAeasy Mini Kit (Qiagen, Hombrechtikon, Switzerland), and reverse transcribed to cDNA using Moloney murine leukemia virus reverse transcriptase (Promega, Dübendorf, Switzerland). Gene specific primers (Microsynth, Balgrach, Switzerland) were designed using a program provided by NCBI (http://www.ncbi.nlm.nih.gov/tools/primer-blast/index.cgi?LINK_LOC=BlastHome), and fitted with BamH1 (forward primers) or HindIII (reverse primers) restriction sites at their 5′ ends, respectively (Table 1). Using these primers and mouse cDNA as a template, specific products were amplified by PCR using Go Taq polymerase (Promega), cut with respective restriction enzymes, and cloned into pBluescript SK+ plasmid (Stratagene/Agilent, Santa Clara, USA). Plasmids encoding mouse tenascin-C and -W cDNAs were obtained from R. Chiquet-Ehrismann (Friedrich Miescher Institute for Biomedical Research, Basel, Switzerland). Digoxygenin-labeled anti-sense and sense RNA probes were generated with a labeling kit from Roche Diagnostics (Koch et al., 1995). The labeled probes were used for *in situ* hybridization as published in detail before (Fluck et al., 2000). In preliminary experiments, serial frontal sections were hybridized with individual probes. All genes described here were found to be equally expressed in the anterior (prospective hard) and posterior (prospective soft) palate, with only minor regional differences (see “Results”).

**Table 1:** **Primers used for generation of gene-specific RNA probes for *in situ* hybridization**.

*Mouse fibrillin-1 (Fbn1: NM 007993.2)*
Forward: 5′CCGGATCCGGGAACCACCAAGGGTGCTG 3′ (nucleotide: 1364–1383)
Reverse: 5′ CCAAGCTTACGCAGTGGAAGCTGCCGTC 3′ (nucleotide: 1626–1606)
PCR product (without restriction sites): 353 bp
*Mouse fibrillin-2 (Fbn2: NM 010181.2)*
Forward: 5′ CCGGATCCCGGTGTGTGGACACCGACG 3′ (nucleotide: 4506–4525)
Reverse: 5′ CCAAGCTTCCCCTCGGCACACTCGTCCA 3′ (nucleotide: 4826–4807)
PCR product (without restriction sites): 277 bp
*Mouse transforming growth factor-β3 (Tgfb3: NM 009368.3)*
Forward: 5′ CCGGATCCCAACCCCAGCTCCAAGCG 3′ (nucleotide: 1578–1597)
Reverse: 5′ CCAAGCTTCCAGGTTGCGGAAGCAGT 3′ (nucleotide: 2046–2026)
PCR product (without restriction sites): 469 bp
*Mouse transforming growth factor-β1 (Tgfb1: (NM 011577.1)*
Forward: 5′ CCGGATCCGTGGACCGCAACAACGCCA 3′ (nucleotide: 1207–1226)
Reverse: 5′ CCAAGCTTGCCGTGAGCTGTGCAGGTG 3′ (nucleotide: 1700–1681)
PCR product (without restriction sites): 488 bp
*Mouse fibronectin: (Fn1: NM 010233.1)*
Forward: 5′ CCGGATCCGACCGAGCCAGGGAGGTGA 3′ (nucleotide: 1211–1230)
Reverse: 5′ CCAAGCTTGAGCTGGGGCACCTCTGGGA 3′ (nucleotide: 1591–1572)
PCR product (without restriction sites): 453 bp

### Immunocytochemistry

A polyclonal rabbit anti-mouse tenascin-W antibody was obtained from R. Chiquet-Ehrismann (Basel, Switzerland) and has been characterized before (Scherberich et al., 2004). Cryosections from paraformaldehyde-fixed mouse embryo heads were blocked with 3% bovine serum albumin in phosphate-buffered saline (BSA/PBS), incubated with anti-tenascin-W (1:100 in BSA/PBS) followed by peroxidase-labeled secondary antibody (Jackson Laboratories; 1:1000 in BSA/PBS). Sections were developed with 0.18 mg/ml chloronaphtol (Merck; stock solution 3 mg/ml in methanol) diluted in PBS, and counterstained with nuclear fast red solution (Sigma, Buchs, Switzerland).

### Microscopy

Slides were viewed with 10× objectives on an Olympus BX-51 microscope. Digital images were recorded using a ProgRes CT3 CMOS camera and ProgRes Capture Pro software (Jenoptik, Jena, Germany). All slides from one experiment were photographed at exactly the same camera settings, and resulting images were processed identically.

### Cell culture and immunoblotting

Cranial fibroblasts were isolated from E14.5 C57BL/6 mouse embryos. The mid-facial region was dissected from embryo heads and incubated in 0.05% trypsin-EDTA solution (Gibco; Invitrogen, Basel, Switzerland) for 1 h at 37°C, after which the tissue was triturated by repeated aspiration. Cells were centrifuged, resuspended in Dulbecco's Minimal Essential Medium (DMEM; Gibco) containing 10% fetal calf serum (FCS; Gibco), plated onto 10 cm cell culture dishes, and incubated at 37°C with 6% CO_2_. At confluency, cranial fibroblasts were passaged by trypsinization. Bmp7 stimulation experiments were performed in 24-well dishes with cells from the second passage. Fibroblasts were first starved for 24 h in DMEM/0.3% FCS, before the medium was changed to DMEM/0.3% FCS containing human recombinant Bmp7 (PeproTech, Hamburg, Germany; 0, 10, 20, 50, and 100 ng/ml, respectively). For positive controls, wells were incubated with DMEM/10% FCS. Cell supernatant was collected from individual wells after 24 h, run on SDS-7.5% polyacrylamide gels under reducing conditions, and proteins were transferred to nitrocellulose. Blots were briefly stained with 0.1% Ponceau Red, and then incubated with rabbit anti-mouse tenascin-W antiserum (see above) or rat anti-mouse tenascin-C monoclonal antibody mTn12 (obtained from Ruth Chiquet-Ehrismann; Aufderheide and Ekblom, 1988), respectively, followed by the matching peroxidase-labeled secondary antibodies (Jackson Laboratories). Blots were developed using ECL reagent (GE Healthcare, Glattbrugg, Switzerland) and viewed on a Storm 840 Phospho-Imager (Molecular Dynamics; GE Healthcare).

## Results

### Correlation of *fibrillin-2* with *Tgf-β3* expression at the time of palatal shelf fusion

**Figure 1:**
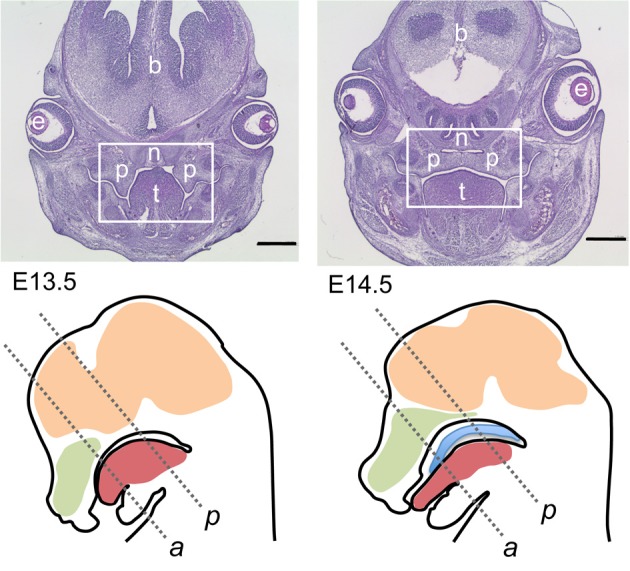
**Overview on mouse sections used for this study. Top row:** Hematoxylin/eosin-stained frontal sections though the heads of E13.5 **(top left)** and E14.5 **(top right)** wildtype embryos; level of posterior palate. The boxed areas correspond to the palatal region depicted in the following figures. At E13.5 the palatal shelves are vertically oriented on each side of the tongue, whereas at E14.5 the shelves have elevated above the tongue and meet at the midline epithelial seam. p, Palatal shelves; t, tongue; n, nasal cartilage; e, eye; b, forebrain. Bar, 600 μm. **Bottom row:** schematic drawings of midsagittal sections through the heads of E13.5 **(left)** and E14.5 **(right)** wildtype mouse embryos. The two frontal section planes used in this study are indicated by dashed lines: *a*, level of anterior palate; *p*, level of posterior palate. Green, nasal cavity; red, tongue; ocre, brain; blue, elevated palate (E14.5).

**Figure 2:**
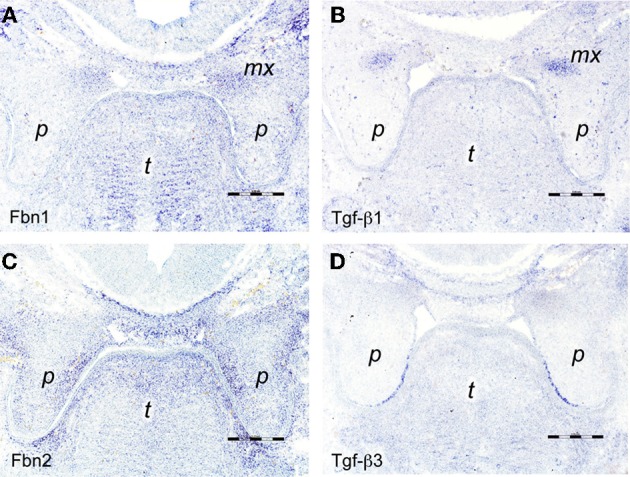
***In situ* hybridizations on frontal sections through E13.5 wildtype mouse embryo heads at the level of the posterior palate with RNA probes specific for (A) *fibrillin-1*, (B) *Tgf-β 1*, (C) *fibrillin-2*, and (D) *Tgf-β3***. The maxillary processes in **(A,B)** show increased expression of *fibrillin-1* and *Tgf-β1* mRNA, respectively. Note enhanced expression of *fibrillin-2* in the proximal-medial mesenchyme of the palatal shelves **(C)**, dorsal of the epithelium positive for *Tgf-β3* mRNA **(D)**. Maxillary process (*mx*), palatal shelf (*p*), tongue (*t*). Bars, 250 μm.

**Figure 3:**
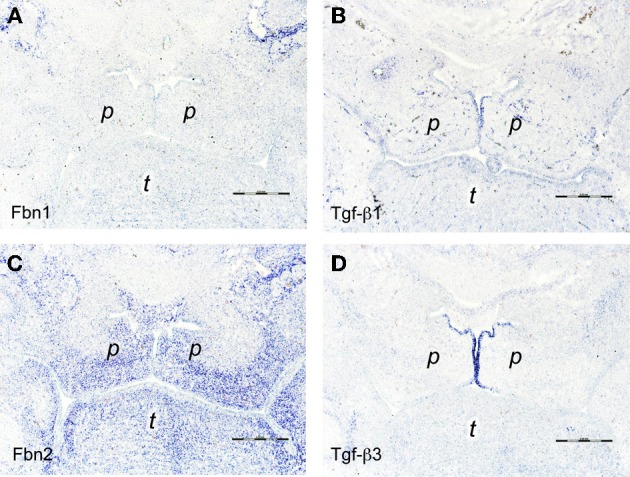
***In situ* hybridizations on frontal sections through E14.5 wildtype mouse embryo heads at the level of the posterior palate with RNA probes specific for (A) *fibrillin-1*, (B) *Tgf-β 1*, (C) *fibrillin-2*, and (D) *Tgf-β3***. Note the strong expression of *fibrillin-2* in the peripheral mesenchyme of the palatal shelves **(C)**, adjacent to the middle edge epithelial cells that express *Tgf-β3* **(D)**. Palatal shelf (*p*), tongue (*t*). Bars, 250 μm.

Tgf-βs are essential for secondary palate formation and fibrillins are known to bind and activate latent Tgf-βs, but the role of fibrillins in palatogenesis has not been investigated. We therefore compared the expression patterns of *fibrillin-1* and *-2* mRNA with those of *Tgf-β1* and −β*3* during palate morphogenesis (see Figure 1 for overview). In E13.5 wildtype embryos, a weak signal for *fibrillin-1* mRNA overlapped with the one for *Tgf-β1* in the developing maxillary processes above the vertically oriented palatal shelves (Figures 2A,B). In contrast to *fibrillin-1*, *fibrillin-2* expression was seen within the vertical palatal shelves themselves, namely in their proximal-nasal mesenchyme (Figure 2C). On the other hand, *Tgf-β3* mRNA was confined to the presumptive medial edge epithelial cells at the distal-nasal aspect of the shelves (Figure 2D). Thus at this stage, only *Tgf-β3* and *fibrillin-2* mRNA appeared to be present within palatal shelves, but no close correlation between their patterns of expression was observed. One day later (E14.5), however, the signal for *fibrillin-2* was much increased in the now horizontally oriented shelves, and it was found everywhere in the peripheral mesenchyme that underlies the palate epithelium (Figure 3C). Thus at this crucial stage immediately before palate fusion, *fibrillin-2* was now expressed in immediate neighborhood to the MEE cells that were strongly positive for *Tgf-β3* (Figure 3D) and more weakly for *Tgf-β1* mRNA (Figure 3B). In contrast to *fibrillin-2*, *fibrillin-1* was not expressed in horizontal palatal shelves (Figure 3A). In conclusion, the data are consistent with the notion that fibrillin-2 expressed in the mesenchyme might be involved in binding Tgf-β3 secreted by MEE cells, and in regulating its activity during palatal shelf fusion.

### Contact-dependent expression of *fibronectin* but not *Tgf-β3* by middle edge epithelial cells

**Figure 4:**
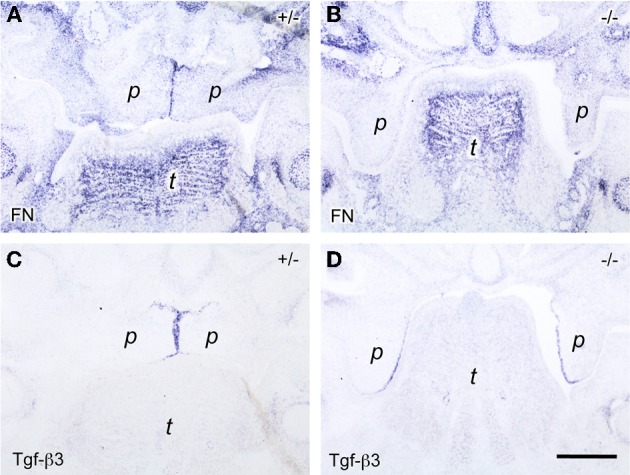
***In situ* hybridization for *fibronectin* (FN; A,B) and *Tgf-β3* (C,D) mRNA, respectively, on frontal sections through the heads of E14.5 *B*mp7^+/−^ (A,C) and *B*mp7^−/−^ (B,D) mouse embryos (anterior level)**. Note *fibronectin* mRNA expressed by midline epithelial cells in the *B*mp7^+/−^ embryo **(A)**, whereas epithelial expression is almost absent in the palatal shelves of the *B*mp7^−/−^ embryo at the same stage **(B)**. Maxillary process (*mx*), palatal shelf (*p*), tongue (*t*). Bar, 200 μm.

Another known regulator of Tgf-β activity is the ECM and cell adhesion protein fibronectin, which was reported to be expressed at the midline before palatal fusion in a Tgf-β3-dependent manner (Martinez-Sanz et al., 2008). We asked whether fibronectin induction in MEE cells in addition required direct contact of palatal shelves. This question was addressed by studying *Bmp7*-deficient mice, which develop cleft palate due to a delay in shelf elevation. In E14.5 wildtype (not shown) or heterozygous *B*mp7^+/−^ embryos (Figure 4A), prominent *fibronectin* mRNA expression was observed at the midline epithelial seam (MES) of opposing shelves, where it overlapped with the *Tgf-β3* signal (Figure 4C). In contrast, little *fibronectin* mRNA was found in the still vertical palatal shelves of E14.5 *B*mp7^−/−^ embryos (Figure 4B), although *Tgf-β3* mRNA was normally expressed in the presumptive MEE cells (Figure 4D; compare to E13.5 wildtype embryo in Figure 2D). Thus, whereas these cells started producing *Tgf-β3* mRNA even before shelves elevate and meet, they expressed *fibronectin* only upon contact of the two shelves at the midline; the latter event failed to occur in *B*mp7^−/−^ embryos. Since, like fibrillins, fibronectin can bind Tgf-βs and modulate their activation, its localized and contact-dependent expression at the midline might be related to the function of Tgf-β3 during palatal shelf fusion.

### Expression of *tenascins* during palatogenesis in wildtype and *Bmp7*-deficient mouse embryos

**Figure 5:**
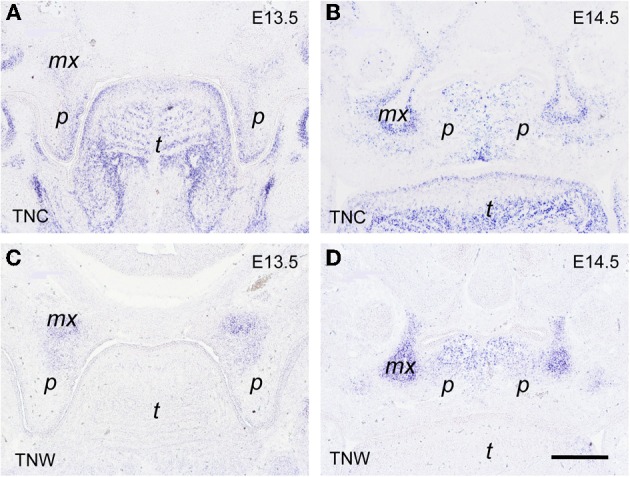
***In situ* hybridization for *tenascin-C* (TNC; A,B) and *tenascin-W* (TNW; C,D) mRNA, respectively, on frontal sections through the heads of E13.5 (A,C) and E14.5 (B,D) wildtype mouse embryos (posterior level)**. Maxillary process (*mx*), palatal shelf (*p*), tongue (*t*). Bar, 250 μm.

Of the four members of the tenascin family of ECM glycoproteins (Chiquet-Ehrismann and Chiquet, 2003), the genes of two are expressed in distinct spatial and temporal patterns during morphogenesis of the secondary palate in the mouse embryo, namely *tenascin-C* and *tenascin-W*, as evidenced by *in situ* hybridization (Figure 5). At E13.5, before elevation of the palatal shelves, *tenascin-C* mRNA accumulated in the mesenchyme close to the nasal and distal surface of the shelf, i.e., underneath the prospective MEE cells (Figure 5A). Accordingly, after shelf elevation at E14.5, *tenascin-C* mRNA was enriched in the palate mesenchyme in a vertical stripe around the MES (Figure 5B). A second area of *tenascin-C* expression was observed at E13.5 near the developing maxillary processes, and at E15 sharply outlined their periphery (Figures 5A,B). In contrast, *tenascin-W* mRNA was detected at both stages within the forming maxillary processes (Figures 5C,D). Weak mesenchymal *tenascin-W* expression was also found in the proximal-nasal quadrant of the vertical shelves at E13.5, and after their elevation this pattern developed into a prominent horizontal stripe in the nasal half of the secondary palate (Figure 5D). Thus at E14.5, tenascin-W positive areas essentially corresponded to the osteogenic domains of the developing palate.

**Figure 6:**
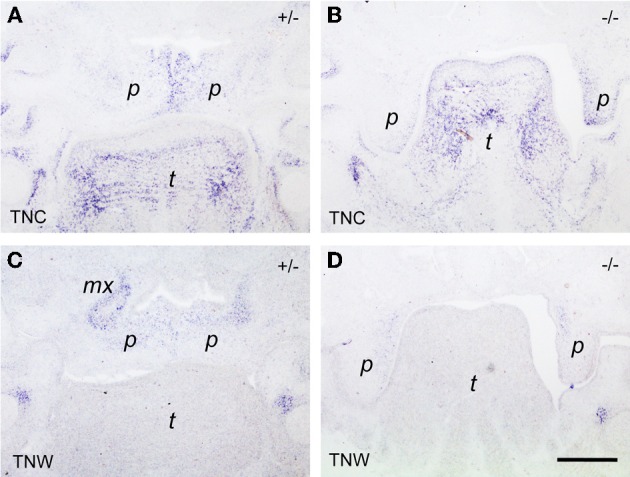
***In situ* hybridization for *tenascin-C* (TNC; A,B) and *tenascin-W* (TNW; C,D) respectively, on frontal sections through the heads of E14.5 *B*mp7^+/-^ (A,C) and *B*mp7^−/−^ (B,D) mouse embryos**. In this anterior section, a low *tenascin-C* signal is seen in the maxillary processes of the heterozygous embryo, whereas expression in the palatal shelves is identical to the posterior level (cf. Figure 5). Note low expression of *tenascin-W* in the palatal shelves of the *B*mp7^−/−^ embryo **(D)** compared to *B*mp7^+/−^ **(C)** or wildtype (cf. Figure 1). Maxillary process (*mx*), palatal shelf (*p*), tongue (*t*). Bar, 200 μm.

Since *tenascin* genes are known to be transcriptional targets of the Tgf-β/Bmp growth factor family, we then asked whether their expression was altered in *Bmp7*-deficient embryos, which develop cleft palate due to delayed shelf elevation. As can be seen from Figures 6A,C, at E14.5 the expression of both *tenascin-C* and *-W* was very similar in the elevated palate of heterozygous *B*mp7^+/−^ embryos when compared to wildtype (cf. Figures 5B,D). In *B*mp7^−/−^ embryos at E14.5 (Figure 6B), *tenascin-C* was found to be expressed in the still vertical shelves in a pattern reminiscent to that of wildtype embryos at E13.5 (cf. Figure 5A). In contrast, *tenascin-W* expression in palatal shelves of Bmp7^−/−^ embryos appeared to be diminished not only relative to heterozygous embryos of the same stage (Figures 6C,D), but also when compared to the vertical shelves of E13.5 wildtype embryos (cf. Figure 5C).

**Figure 7:**
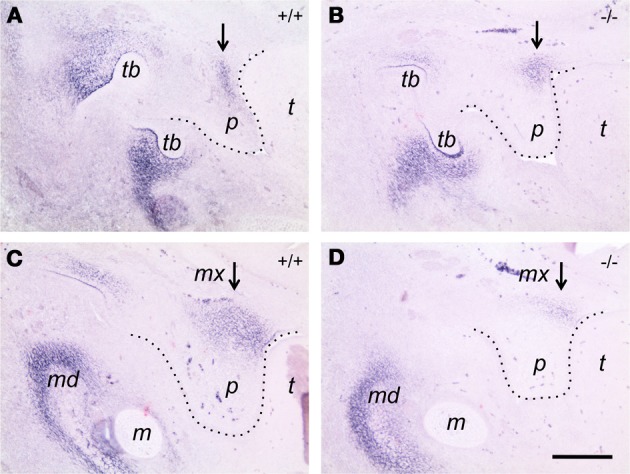
**Immunocytochemistry for tenascin-W on frontal sections of E13.5 *B*mp7^+/+^ (A,B) and *B*mp7^−/−^ (C,D) embryos**. Sections through the anterior **(A,C)** and the posterior **(B,D)** palate are shown. Note tenascin-W protein staining extending into the palatal shelf in the *B*mp7^+/+^ embryo **(A)**, whereas it remains focussed to the region of the maxillary process in the *B*mp7^−/−^ embryo **(B)**. In the posterior palate, tenascin-W protein expression is reduced in the maxillary process of the *B*mp7^−/−^ **(D)** compared to the *B*mp7^+/+^ **(C)** embryo (arrows). *mx*, Maxillary process; *p*, palatal shelf; *t*, tongue; *tb*, tooth bud; *md*, mandible; *m*, Meckel's cartilage. Bar, 200 μm.

To investigate a possibly aberrant expression of *tenascin-W* in palatal shelves of *Bmp7*-deficient embryos also on the protein level, we compared frontal sections from control and *Bmp7*-deficient E13.5 embryo heads (i.e., before palate elevation in the wildtype) by immunohistochemistry. Tenascin-C protein distribution turned out to be similar in palatal shelves from heterozygous versus *B*mp7^−/−^ embryos at both the anterior and posterior level at this stage (not shown), and it fitted with the mRNA expression pattern observed previously by *in situ* hybridization (cf. Figure 5). In contrast, tenascin-W protein was distributed differently in the anterior palate of E13.5 *B*mp7^−/−^ embryos when compared to the wildtype control (Figures 7A,B), and in the posterior palate its expression was considerably reduced in the maxillary process (Figures 7C,D). These findings are in accordance with the hypothesis that *tenascin-W* (but not *tenascin-C*) might be a downstream target of Bmp7 associated with palatal shelf reorientation.

### Induction of *tenascin-W* but not tenascin-C by Bmp7 in embryonic cranial fibroblasts *in vitro*

**Figure 8:**
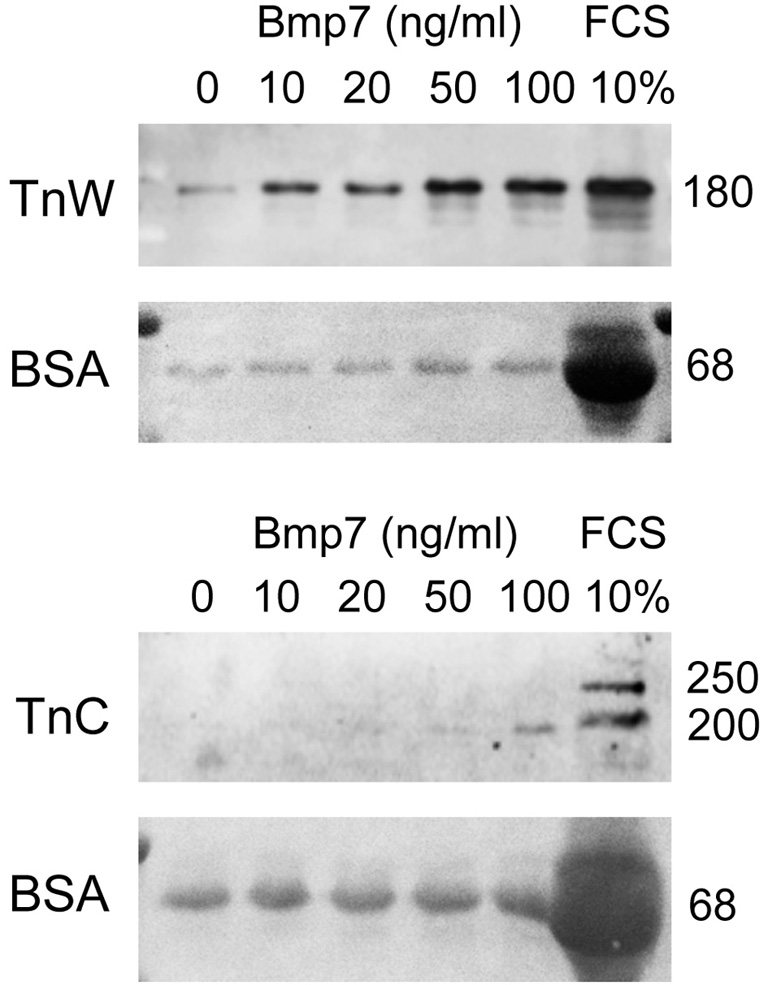
**Immunoblotting for tenascin-W (TnW) and tenascin-C (TnC) in media conditioned by mouse embryonic cranial fibroblasts, which were stimulated with the indicated concentrations of Bmp7 for 24 h**. The last lane of each blot shows a control culture stimulated with 10% fetal bovine serum (FCS). Below each immunoblot, Ponceau Red staining of the corresponding membrane is shown; medium-derived bovine serum albumin (BSA) was used for loading control. Numbers at right indicate protein molecular weights in kDa.

Since we observed an altered pattern of expression for tenascin-W in the palatal region of Bmp7^−/−^ embryos, we asked whether this ECM protein was a direct target of Bmp7 signaling. To this aim, cranial fibroblasts were isolated from the heads of E14.5 wildtype embryos, and cultured under low serum conditions in the presence of increasing concentrations of recombinant Bmp7 (rBMP7). For negative and positive controls, cells were grown in either low (0.3%) or high (10%) fetal calf serum, respectively. The medium was collected after 24 h, and secreted tenascin-C and -W protein was analyzed by immunoblotting. As is evident from Figure 8, already 10 ng/ml rBMP7 strongly stimulated tenascin-W (180 kDa) production by cranial fibroblasts compared to the low serum control; maximal induction was seen with 50 ng/ml. In contrast, 100 ng/ml rBMP7 were required to detect a weak signal for the small (200 kDa) but not the large (250 kDa) splice variant of tenascin-C. Secretion of both tenascins was strongly induced by 10% serum (Figure 8). Thus, Bmp7 appears to directly regulate the production of tenascin-W but not tenascin-C, which agrees with the results obtained from the *B*mp7^−/−^ embryos.

## Discussion

### Interaction between growth factors and extracellular matrix during palatogenesis

Cleft lip, with or without cleft palate, and isolated cleft palate occur with a frequency of 1–2 in thousand live births. They represent the most common craniofacial birth defects in humans (Mitchell, 2009). Genetic and environmental factors cause these congenital anomalies (Jugessur and Murray, 2005). It has been noted that various failures of either palatal shelf growth, elevation, adhesion, or the lack of mesenchymal differentiation and disappearance of the MES can cause cleft palate (Gritli-Linde, 2007). Thus, the study of palatogenesis and the etiology of cleft palate are key to understanding oral malformations. The essential roles of Tgf-βs and Bmps during palatogenesis have been investigated by various authors in the developing mouse embryo (Proetzel et al., 1995; Martinez-Alvarez et al., 2000; Nawshad et al., 2004; Zouvelou et al., 2009a). It is well established that it is a major function of these growth factors to regulate ECM synthesis and turnover (Verrecchia and Mauviel, 2007; Tucker and Chiquet-Ehrismann, 2009). However, it is completely unknown so far whether changes in the expression of specific ECM components might be causally involved in the craniofacial and other malformations observed in *Tgf-β–* or *Bmp*-deficient mice. In other words, maybe with the exception of the well-established function of Tgf-β3 during palatal fusion, it is still largely a mystery how a growth factor deficiency eventually translates into a specific congenital malformation. Since ECM proteins are transcriptional targets of Tgf-β/Bmp growth factors and essential for tissue structure, as well as for cell adhesion and migration, it is obvious to hypothesize that they might be causally involved in linking growth factor deficiency to CLP.

### Why study tenascins and fibrillins in the context of palatogenesis?

Although numerous extracellular proteins are expressed in the developing orofacial complex, we have so far focused our attention mainly on two families of ECM glycoproteins, namely tenascins and fibrillins. Tenascins belong to the so-called “matricellular” ECM proteins that modulate cell-ECM interactions and ECM assembly (Chiquet-Ehrismann and Chiquet, 2003). Of the four family members, two are expressed in the developing secondary palate, namely tenascin-C and tenascin-W. In contrast to the prototype adhesive ECM protein fibronectin, both these tenascins are “anti-adhesive” in a context-dependent manner, i.e., they interfere with fibronectin-mediated cell spreading (Chiquet-Ehrismann et al., 1988; Brellier et al., 2012). Thus, they might be involved in tissue reorganization during palatogenesis. Fibrillins, on the other hand, are cysteine-rich glycoproteins present in extracellular microfibrils in elastic and nonelastic tissues (Keene et al., 1991; Ramirez and Sakai, 2010). These ECM components not only play a structural role in integrating connective tissues, but they also have an important regulatory function in modulating the distribution and activation of growth factors of the Tgf-β/Bmp family, which in turn regulate ECM formation and cell differentiation (Ramirez and Rifkin, 2009). Mutations in the *Fibrillin-1* gene result in a complex connective tissue disorder called Marfan syndrome with prevalent cardiovascular, ophthalmologic and orthopaedic signs (Lee et al., 1991). These mutations also cause dysregulation of Tgf-β/Bmp activation and signaling (Nistala et al., 2010), resulting in apoptosis in the developing lung (Neptune et al., 2003). Mutations in *Fibrillin-2* are responsible for a related, but less severe condition, namely congenital contractural arachnodactyly with mostly musculoskeletal manifestations (Lee et al., 1991; Ramirez and Rifkin, 2009). It is therefore reasonable to believe that fibrillins modulate the activity of Tgf-β/Bmp growth factors also during craniofacial morphogenesis.

### Fibrillin-2 and fibronectin: modulators of Tgf-ß3 activity during palatal shelf fusion?

Growth factors of the Tgf-β family are secreted as inactive complexes with latent Tgf-β binding protein (LTBP), which itself can bind to fibrillin or fibronectin microfibrils (Ramirez and Rifkin, 2009). Activation of ECM-bound, latent growth factor complexes involve their interaction with integrins on cell surfaces and the application of cellular force (Wipff et al., 2007). In the complex process of activation, fibrillins (Ramirez and Rifkin, 2009) and fibronectin (Fontana et al., 2005) play an important role by presenting latent growth factor complexes to cells, thereby controlling bioavailability of active Tgf-βs. Interestingly, it has been shown recently that fibrillin-1 and fibrillin-2 regulate Tgf-β and Bmp activity in distinct ways: Whereas both fibrillins stimulate Tgf-β activation, binding of Bmps to fibrillin-1 (but not fibrillin-2) reduces activity of the latter growth factors (Nistala et al., 2010). It is therefore important to know how *fibrillin-1* and *-2* are expressed during palatogenesis in relation to growth factors known to be involved in this morphogenetic process. Here we show that *fibrillin-1* expression decreases whereas that of *fibrillin-2* increases during palatogenesis. This observation fits with the notion that both Tgf-βs and Bmps need to be activated during the process. In addition, we found that *fibrillin-2* was expressed in the mesenchyme adjacent to the MES before palatal shelf fusion. Moreover *fibronectin*, which is also known to positively regulate Tgf-β activation (Fontana et al., 2005), was expressed by MEE cells upon their contact. Thus, *fibrillin-2* and fibronectin might be important for the essential role of Tgf-β3 during palatal shelf fusion. This hypothesis could be tested, e.g., in tissue culture, by examining the fusion capacity of *fibrillin-2/Tgf-β3* double deficient palatal shelves in the presence or absence of Tgf-β3. Alternatively, it would be interesting to test whether heterozygous *Tgf-β3*^+/−^ embryos (which develop normally) would present with cleft palate on a *fibrillin-2^−/−^* background.

### Tenascin-W: a possible downstream target of Bmp7 during palate reorientation?

Because of its region-specific expression in embryonic palatal shelves before and after their reorientation, tenascin-C has been implicated in morphogenesis of the secondary palate more than 20 years ago. Since then, however, *tenascin-C* knockout mice have been generated, and these do not exhibit overt craniofacial abnormalities (Saga et al., 1992; Forsberg et al., 1996). A possible explanation for the surprisingly mild phenotype of *tenascin-C*-deficient mice might be compensation by other ECM proteins. A likely candidate is the functionally related *tenascin-W* (gene name *Tnn*), whose expression is similar to that of *tenascin-C*, especially in osteogenic regions of the embryo (Scherberich et al., 2004). In the mouse head, *tenascin-W* expression starts in the maxillary processes at E13.5 (Scherberich et al., 2004), and we show here that *tenascin-W* mRNA and protein is detected in a distinct spatial-temporal pattern during development of the secondary palate. In E13.5 wildtype embryos, *tenascin-W* is found in the vertical palatal shelves anteriorly in the nasal mesenchyme, and more posteriorly in the ossifying region of the maxillary processes. After shelf reorientation, *tenascin-W* is expressed in the dorsal half of the secondary palate, partially (but not completely) overlapping with *tenascin-C*. In *B*mp7^−/−^ mice in which palatal shelf reorientation is delayed, tenascin-W (but not tenascin-C) protein appeared to be distributed differently in the anterior shelf, and its amount was reduced more posteriorly. In perfect agreement with these results, the secretion of tenascin-W but not tenascin-C protein was found to be induced by recombinant Bmp7 in cranial fibroblasts isolated from E14.5 mouse embryos. We therefore speculate that *tenascin-W* might be a downstream effector of Bmp7 signaling, and that cleft palate in the *Bmp7* knockout mouse might in part be caused by a misexpression of *tenascin-W*. To establish a causal relationship, however, it needs to be tested whether Bmp7, like Bmp2 (Tucker and Chiquet-Ehrismann, 2009), regulates *tenascin-W* at the transcriptional level, and whether *tenascin-W-deficient* mice exhibit a palate phenotype. These experiments are underway.

## Conclusions and perspectives

The present study was undertaken to obtain first evidence for a functional interaction between Tgf-β/Bmp growth factors and selected ECM glycoproteins during morphogenesis of the secondary palate in mammalian embryos. Based on our results, we speculate that misexpression of the “matricellular” ECM protein tenascin-W might be involved in causing the cleft palate phenotype of the *Bmp7* knockout mouse, and that fibrillin-2 and fibronectin could be important for the activation of Tgf-β3 at the palatal midline during fusion. Our present findings provide the basis for testable hypotheses concerning the precise function of ECM components as downstream effectors of growth factor signaling during palatogenesis.

### Conflict of interest statement

The authors declare that the research was conducted in the absence of any commercial or financial relationships that could be construed as a potential conflict of interest.
